# What Is the Role of Local Antimicrobial Protection for One-Stage Revision for Peri-Prosthetic Hip Infection?

**DOI:** 10.3390/antibiotics13111060

**Published:** 2024-11-07

**Authors:** Carlo Luca Romanò, Luigi Bonomo, Giulio Bonomo, German Viale, Hernán Del Sel, Mohammad Tezval

**Affiliations:** 1 Romano Institute, 1001 Tirana, Albania; 2 Universitatea de Medicina si Farmacie Victor Babes din Timisoara, 300041 Timișoara, Romania; lbonodoc@gmail.com (L.B.); gbonodoc@gmail.com (G.B.); 3 Department Orthopaedics and Traumatology, British Hospital of Buenos Aires, Buenos Aires 1280, Argentina; gjviale@gmail.com (G.V.); hdelsel@argentina.com (H.D.S.); 4 Klinikum Vest GmbH, Dorstener Str. 151, 45657 Recklinghausen, Germany; mohammad.tezval@klinikum-vest.de

**Keywords:** hip, infection, prosthesis, PJI, one stage, single stage, revision, review, local antibiotics, antibacterial coatings

## Abstract

The aim of this review is to investigate the effective role of local antimicrobial protection for one-stage cemented and cementless hip revision surgery. Twelve studies reporting the results of cemented single-stage procedures with a minimum two-year follow-up were reviewed. When pooling together the data, no infection recurrence was observed on average in 83.3% of the patients (a range of 75.0% to 100%). Only two papers included patients treated without the use of antibiotic-loaded bone cement, with an average infection control of 95.9% in a total of 195 patients. This figure appears to be better than the 80.7% infection control obtained by pooling together all the remaining studies. Concerning cementless one-stage revision, a total of 17 studies, reporting on 521 patients, showed an average of 90.0% (range 56.8% to 100%) no infection recurrence at a minimum two-year follow-up. No comparative study investigated cementless revision with or without local antibacterial protection. The pooled data showed an average infection control of 86.7%, without the application of local antibacterials, compared to 90.1% to 100% with local antimicrobial protection, depending on the technology used. No statistical difference could be found, either considering local antibacterial strategies alone or pooled together. No side effects had been reported by any local antibacterial technique. Local antibacterial protection for one-stage hip revision surgery, although safe and largely performed in the clinical setting, appears to still rely mainly on experts’ opinions with no prospective or comparative trial, hence no definitive conclusion can be drawn concerning its effective role in one-stage hip revision surgery.

## 1. Introduction

*“Rates of peri-prosthetic joint infection (PJI) in primary total hip and total knee arthroplasty range between 0.3% and 1.9%, and up to 10% in revision cases. Significant morbidity is associated with this devastating complication, the economic burden on our healthcare system is considerable, and the personal cost to the affected patient is immeasurable”* [1]. 

The occurrence of peri-prosthetic joint infection (PJI) generally requires the removal of the infected implant and its exchange in a single- or two-stage surgical procedure. The operative approach is determined by a combination of the surgeon’s experience, clinical and radiological presentations, and available bone stock and infection factors, with the majority of surgeons opting for a two-stage procedure, which has been traditionally believed to be more secure and successful [2,3]. On the other hand, a one-stage approach does offer self-evident advantages over a staged procedure, including reduced hospitalization, costs, and time to recovery [4]. Moreover, several recent studies and systematic reviews have pointed out the lack of a statistically significant difference in infection recurrence rates after one-stage or two-stage hip revision surgery [5,6]. These observations are progressively prompting more and more surgeons to propose one-stage strategies to their patients and novel one-stage techniques have been proposed in recent years.

In fact, the first and the most often reported one-stage technique requires the fixation of the new implant with antibiotic-loaded bone cement, which is considered a key step for the success of the procedure [7]. On the other hand, more recently, various authors reported that cementless revision hip prostheses, with or without the application of local antibiotic delivery systems, can be equally effective [6].

The aim of the present review of the current literature is to investigate the effective role of local antibacterial protection technologies for one-stage cemented or cementless hip revision surgery and test the hypothesis that local antibiotic implant protection may have a positive impact on reducing the infection recurrence rate after this surgery. For the purpose of the present analysis, “local antibacterial protection technologies” are considered all the coatings, delivery systems, or implant surface modifications intended to provide local delivery of antibiotics and/or to reduce/prevent bacterial adhesion and biofilm formation. Moreover, cemented or cementless hip revision surgery indicates the reimplantation of the hip joint prosthesis with an implant fixed, respectively, with or without the local application of polymethylmethacrylate bone cement.

## 2. The Role of Local Antibacterial Protection in Cemented One-Stage Hip Revision Surgery

In the early 1970s, Dr. Hans Wilhelm Buchholz conducted extensive research on antibiotics and polymethylmethacrylate (PMMA) in the context of hip and knee replacement surgery. He consistently reported lower infection rates with the addition of gentamycin antibiotics to the bone cement. Dr. Buchholz was among the first to demonstrate the successful use of antibiotic-loaded bone cement for preventing infections in endoprostheses, as well as using single-exchange arthroplasty for treating infected prostheses [8]. One-stage exchange arthroplasty, using techniques and principles similar to those originally described by Buchholz’s team at the ENDO Klinik in Hamburg, Germany, has since been adopted by various centers around the world [9,10,11,12].

The three key principles of the ENDO Klinik have been well described and recently reconfirmed [13,14]. First, the organism must be identified along with its sensitivities and minimum inhibitory concentrations. According to its original description, single-stage revision should not be performed without this information as antibiotic treatment cannot be appropriately tailored to combat the infection. Joint aspiration is hence performed with the patient off antibiotics for at least 14 days, using an “as sterile as possible” technique with a culture incubation period of 14 days. The second principle is debridement. Aggressive debridement and complete removal of all infected tissues and implanted biomaterials is considered a pivotal step for the success of the technique. The third principle involves both local and systemic antibiotic delivery tailored to the identified pathogenic organism. Local antibiotic delivery is achieved through cement [15]. According to the authors that first described this method, PMMA ensures much higher tissue concentrations at the infection site than systemic administration. Bactericidal antibiotics, such as aminoglycosides, cephalosporins, fluoroquinolones, metronidazole, penicillin, and vancomycin, can be advantageously mixed, while some authors also consider clindamycin an acceptable bacteriostatic option [5]. Up to 10% of the dry crystalline weight of antibiotics can be added to the cement without significant mechanical loss. 

Concerning safety, while local antibiotics achieve high intra-articular concentrations with lower systemic risks, there are rare case reports of systemic complications like renal or hepatic failure and allergic reactions [16]. However, pharmacokinetic studies investigating antibiotic concentrations released from PMMA showed serum and urine concentrations below toxic thresholds [17,18,19,20], while the local cytotoxicity of eluted antibiotics demonstrated good cell survival/recovery capacities after high antibiotic concentration exposure for antibiotics such as cephazolin, vancomycin, and aminoglycosides [21,22], even if high local levels of gentamicin have been shown by some authors to have a detrimental impact on osteogenesis [23]. Another big concern about prolonged local antibiotic delivery from bone cement is the development of microbial resistance. This has been disproven by several authors [24,25,26,27]. Overall, the potential toxic effects and risks of the local release of antibiotics by bone cement are considered extremely low and rare and do not prevent the current widespread use of antibiotic-loaded bone cement in various clinical settings [28,29,30]. On the other hand, it should be emphasized that the presence of antibiotics in bone cement has been found able to reduce bacterial biofilm formation, but it may not completely inhibit its presence [31].

The scientific background of the clinical use of local antibiotics released from bone cement relies on in vitro [32,33] and in vivo studies [34,35]. Moreover, comparative clinical trials, investigating low- or high-dose local antibiotics delivery and/or single versus dual antibiotics, do bring evidence that higher doses or combinations of antibiotics improve post-surgical infection control compared to lower doses or single antibiotics. Jenny and co-workers reported on a prospective, single-center clinical trial showing a statistically significant 50% reduction in the infection recurrence rate after one-stage hip or knee revision surgery using high-dose gentamycin and clindamycin-loaded bone cement compared to a low dose [36]. Similarly, Szymski et al. recently reported data from the German register, showing better infection prevention after hip prosthesis for femoral neck fracture management by using dual antibiotics in bone cement compared to a single antibiotic [37]. This study is in line with and confirms a previous observation made in the United Kingdom [38]. However, to the best of our knowledge, there is a lack of prospective studies or systematic reviews and meta-analyses comparing local antibiotic delivery from PMMA to plain cement for the one-stage treatment of peri-prosthetic hip infection. 

We performed a thorough and comprehensive literature search of studies fully written in English (or with an abstract in English) on cemented and uncemented one-stage hip revision surgery for delayed periprosthetic hip infection by searching the following internet databases: EMBASE; PubMed/Medline; Medline Daily Update; Medline In-Process and other non-indexed citations; Google Scholar; SCOPUS; CINAHL; Cochrane Central Register of Controlled Trials and Cochrane Database of Systematic Reviews; and NHS Health Technology Assessment; http://www.google.com. We used the following keywords either alone or in a variety of combinations: hip; infection; arthroplasty; prosthesis; total hip replacement; THR; THA; prosthetic hip infection; periprosthetic hip infection; exchange arthroplasty; one-stage; single-stage; cemented; cementless; and uncemented. The results of all studies that included five or more cases and had a minimum follow-up of 24 months are reported in Table 1 and Table 2.

Means and standard deviations (SDs) were calculated on pooled data and compared. Statistical analysis was performed using *t*-tests and Fisher’s Exact test where appropriate. A *p*-value of <0.05 was deemed to be statistically significant.

Pooling together the data of the 12 studies available for our analysis reporting on delayed (>6 weeks from surgery) PJI, the average success rate of one-stage antibiotic-loaded cemented hip revision is 83.2% (ranging from 75% to 100%) (cf. Table 1).

Local antibiotic(s) administration through bone cement appears to be the preferred choice of the majority of authors reporting one-stage hip revision surgery. Seven out of the twelve studies included in our analysis disclosed a selection bias, as patients with draining fistula, unknown or multi-resistant pathogen(s), or immunocompromised hosts were excluded (cf. Table 1).

We found only three clinical trials reporting cemented one-stage revision surgery without the use of local antibiotics. All those studies were performed in France. Klouche and co-workers were the first to publish a striking 100% infection eradication rate at a two-year minimum follow-up in a series of 38 patients [45]. In line with this, a few years later, Zeller et al., in a large multi-center cohort study on 157 patients treated with one-stage exchange arthroplasty and twelve weeks of systemic antibiotics and no antibiotics in the cement, showed only two relapses and six new infections, with a cumulative infection control rate of nearly 95% at five years postoperatively [10]. In a more recent study, conducted in the same center [64], an overall infection eradication rate of 95.3% at a minimum two-year follow-up was reported in a series of 66 patients treated either with cemented (*n* = 21) or cementless revision prostheses (*n* = 45), without the addition of local antibacterials. Moreover, all of these patients had a fistula at the time of surgery, actively draining in 76% of cases, a condition that is considered by many to be a bad prognostic factor and even a contraindication to a one-stage procedure [65] (this study is not included in Table 1, as it does not distinguish between hip and knees).

When pooling together the data of the French studies from Klouche and Zeller (*n* = 195) and comparing them with the results reported by all other authors (*n* = 1013), the difference in average infection control, 95.9% versus 80.7%, is unexpectedly extremely statistically significant in favor of no local antibiotic administration (*p* < 0.0001). Similar results are obtained even when excluding the oldest papers published before 1995. In this case, no infection recurrence is observed in 88.9% of 180 patients, a value that is still statistically inferior to that of Klouche and Zeller (*p* = 0.01).

While this comparison has obvious methodological limits as it considers different patient populations across various centers, with possible bias and heterogeneous material, it adds to the fact that, on the basis of the available literature, there is no clinical evidence that delivering local antimicrobials through PMMA is necessary in single-stage hip revision surgery. Hence, more than four decades after its first description, the use of antibiotic-loaded bone cement appears to remain largely based on experts’ opinions and data obtained in pre-clinical studies or comparative clinical trials in other settings [66].

## 3. The Role of Local Antibacterial Protection in Cementless One-Stage Hip Revision Surgery

For various reasons, cementless fixation of hip implants is currently the predominant choice of surgeons, according to the UK National Joint Register [https://reports.njrcentre.org.uk/AR-Executive-Summary, accessed on 20 October 2024] and the Swedish register [67], even if it was not proven to be superior to cement in a recent systematic review [68]. 

Cementless one-stage revision to treat peri-prosthetic hip infection has also been gaining more and more acceptance worldwide in recent years. In fact, the largest series on cemented one-stage hip revision surgery dates back up to four decades ago, while in the last twenty years, a growing number of papers reporting on cementless one-stage reimplantation have been found (cf. Table 2).

Seventeen observational studies reporting on delayed peri-prosthetic hip infection treated with cementless one-stage revision, for a total of 521 patients, were retrieved by our search. No randomized, prospective controlled trials comparing different cementless one-stage techniques could be found. The majority of papers described retrospective series without a control group (Level of evidence: IV). The number of cases ranged from 5 to 111 (mean 30.6 ± 25.0). Overall, at an average follow-up of 65.5 ± 22.5 months (range 24 to 78 months), infection control was obtained in 90.0% of the cases (ranging from 56.8–100%) (Table 2). 

Eight studies reported one-stage cementless revision without the use of local antibiotics. Among these, five reported a selection bias, excluding patients with open fistulas and/or severe bone loss and/or unknown or multi-resistant pathogen(s) or immunocompromised hosts. Moreover, Bori et al. [52] and Born et al. [54] did report the use of antibiotic-loaded cemented cups in some cases, while Yoo et al. [50] performed only cup revision in four patients out of the twelve treated. When pooling the results of all studies reporting no local antibiotic administration (*n* = 181), an average infection control of 86.7% (ranging from 56.8–100%) at a mean follow-up of 80.3 ± 23.4 months can be calculated (cf. Table 3).

The remaining nine studies reported the results of four different local antibiotic delivery techniques, which, on average, provided the following no-infection-recurrence rates: intra-articular post-operative antibiotic infusion or local antibiotic vancomicin powder application at the time of surgery: 90.1%; antibiotic-loaded collagen-fleece: 91.1%; antibiotic-impregnated allografts: 93.5%; and antibiotic-loaded hydrogel coating: 100% (cf. Table 3). Three studies excluded patients with open fistulas, unknown or multi-resistant pathogen(s), or immunocompromised hosts (cf. Table 2). No side effects were reported with the use of any local antibacterial technology. Due to the relatively low number of patients, no statistical difference could be demonstrated (*p* > 0.05) by single or pooled local antimicrobial protection systems compared to no local antibiotic application.

One study [69] reported the results of single-stage cementless revision surgery without local antibiotic delivery for the treatment of early infections (<6 weeks after implant); this report showed remarkably low infection control (56% or 15/27 patients) at a mean follow-up of 50 months (range, 27–89 months). This observation compares to a similar study conducted by Riemer and co-workers that, with the use of gentamicin-loaded collagen fleece, reported successful implant retention at a mean follow-up of 60 months in 18/18 patients [70].

## 4. Discussion

In this review, we addressed the relative role of antibacterial local protection in one-stage cemented or cementless revision surgery for a peri-prosthetic hip infection. 

While this analysis does confirm previous observations concerning cementless one-stage hip revision success rates similar or even superior to that of cemented implants [6,14,71], the reviewed data do not conclusively support the need for local antibiotic delivery for periprosthetic hip infection control.

In particular, our findings challenge the superiority of antibiotic-loaded cemented one-stage hip revision surgery and contradict the traditional prescription of antibiotic-loaded bone cement as a key factor in performing successful one-stage hip revision surgery [8,72]. The lack of comparative trials is mainly due to the French experience, reported by Klouche, Zeller, and co-workers [10,45]. Upon further analyzing those data in light of the current knowledge regarding biofilms and bacterial adhesion capabilities, the limit of bone cement as a local antibacterial implant protection system appears evident. In fact, antibiotic-loaded bone cement is only applied to the interface between the cup and/or the stem of the prosthesis, leaving all the extramedullary and the modular parts of the implant unprotected. This technical limitation may not be overcome unless antibiotic-loaded bone cement is used in combination with other technologies also able to protect the uncemented parts of the prosthesis. Moreover, not all antibiotic-loaded bone cements are the same or provide the same antibiotic elution. Manual mixing of antibiotics with PMMA has been shown to provide a significantly different release of various antibacterial agents compared to pre-manufactured antibiotic-loaded bone cement [73]. The use of single antibiotics or a combination of several, their relative concentration in bone cement, and the porosity of PMMA are some of the many factors that come into play and determine the effective pharmacokinetics of the local antibiotic administration [74,75]. Finally, biofilms and small-colony variants of bacteria have been retrieved in antibiotic-loaded bone cement [76,77], demonstrating the ability of bacteria to overcome even local antibiotic protection if conditions favorable to their persistence are met. The lack of a standard for local antibiotic administration through bone cement may well explain, among other factors, the wide range of results reported in the literature. 

**Figure 1:**
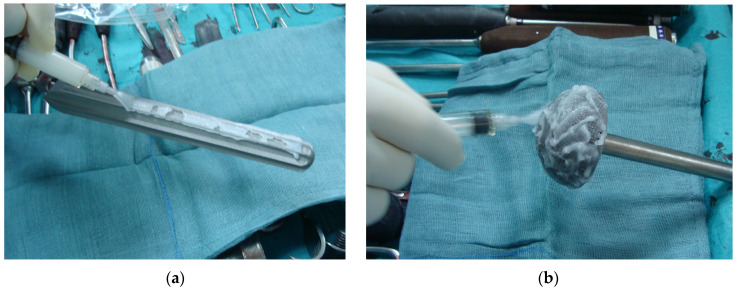
Application of the DAC antibiotic-loaded hydrogel coating to (**a**) a cementless hip stem revision prosthesis and (**b**) an acetabular cup.

On the other hand, our review also reveals that one-stage cementless hip revision surgery with local antibacterial protection has not been proven more effective than that without in comparative clinical trials. Moreover, when pooling together the results of single or combined local antibacterial protection strategies, no statistically significant difference in infection control can be demonstrated compared to one-stage cementless hip revision without the use of local antibacterials. However, our results concerning the efficacy of local antibacterial protection in this setting should be interpreted with caution due to the many limitations of the material available for our analysis. First of all, the number of patients treated with each local antibacterial modality is quite low and heterogeneous, thus reducing the validity of the comparison of results. Additionally, pooling together the results of the patients treated with different local antimicrobial strategies can be questionable as those chosen by different authors were completely different from one another concerning both the type of treatment and the site of application. As an example, while the antibiotic-loaded hydrogel coating may be applied to all the implant surfaces and components (cf. Figure 1), vancomycin powder, local antibiotic irrigation, and other technologies may not. This may have a strong impact on the final outcome as it is well known that the primary step in bacteria colonizing an implant is attaching to the inert surfaces of the biomaterials and immediately starting to form biofilms [78]. 

Several in vivo and clinical studies have shown that if the implant surface is effectively protected by an antibacterial coating, it is extremely effective in preventing implant-related infection development [79,80,81]. Moreover, while most of the proposed solutions to provide local antimicrobial protection act at the time of surgery, others like local antibiotic irrigation may only take effect after surgery when the bacteria eventually present in the surgical field have had time to attach to the implant and hence become difficult to reach using intra-wound irrigation. 

Moreover, it is worth noting that five out of the eight studies reporting on one-stage treatment without local antibacterials were performed on selected patients with a less severe infection, minor bone involvement, and better hosts. In the only direct comparison between a single- and two-stage exchange, Wolf et al. [51] showed mean infection control exceeding 96% after two-stage treatment compared to less than 57% after cementless single-stage treatment without the use of local antibiotic protection. Further analyzing the data, the authors provided evidence that the difference between the two treatments was due to the better results obtained with a two-stage approach in more compromised hosts, while one-stage and two-stage treatments did perform equally well when normal hosts and early infections were involved. Selection bias is also a well-known limitation when comparing the results of one-stage procedures with two-stage procedures as many authors prefer a staged approach to manage the most complicated cases, and inevitably, most of the retrospective series reported on one-stage treatment include less severe patient populations [82]. 

Another limitation of the present review is that it did not explore how patient factors such as co-existing conditions, age, body mass index, gender, type of implant, prior surgeries, etc., might influence the outcomes. It also did not assess different surgical approaches including the type of hip revision prosthesis, the surgical technique, or the need for bone grafts. The role of the pathogen(s) and its antibiotic resistance profile was also not investigated as that of systemic antibiotic administration and the use of single or dual local antibiotics. Furthermore, our analysis did not distinguish between cases of infection recurrence due to the same or a different pathogen. Finally, the comparison of historical studies with the most recent ones can be biased as the diagnostics and even the definition itself of periprosthetic joint infection have evolved and changed several times over the years.

Its main limitations notwithstanding, this review suggests that single-stage exchange arthroplasty is a viable option for the treatment of chronic periprosthetic hip infections. Local antibacterial treatment is safe, even if its superiority over no local antimicrobial protection is not proven. The fact that, with the current data available, is not possible to prove the clinical benefit of local antibacterial protection for one-stage hip revision surgery for the treatment of peri-prosthetic infection has a clear impact on research, clinical, and medico-legal aspects. This also may ground the ethical basis for designing prospective comparative studies with and without local antibacterial protection. In fact, the limitations and biases in the current literature underscore the need for standardized reporting methods, particularly in the description of local antimicrobial techniques and microbial identification and antibiotic resistance, while large-scale, multicenter, prospective, randomized trials may allow us to definitively determine the real impact of local antibacterial implant protection, if any. Even if logistical challenges such as the low incidence of the disease, small patient populations, long-term follow-up requirements, and variations in microorganisms make conducting such studies exceptionally difficult, such studies are possible and have been performed successfully in other clinical settings, provided that multicenter trials are well designed and properly funded [83,84].

## Figures and Tables

**Table 1:** Data from studies reporting cemented one-stage hip revision surgery for delayed (>6 weeks after surgery) peri-prosthetic hip infection, at a minimum follow-up of 24 months.

Author	Year	Number of Patients	Number of Patients Free from Infection at Follow-Up	Percent of Patients Free from Infection at Follow-Up	Follow-Up (Months)			Selection Bias	Local Antimicrobial Protection
					Min	Max	Mean		
Buchholz [39]	1981	582	448	76.8	24	132		No	Yes
Miley [40]	1982	46	40	87	32		48.5	Yes	Yes
Wroblewski [9]	1986	102	93	91.2	38.8			No	Yes
Sanzen [41]	1988	102	77	75.5	24	108		No	Yes
Raut [42]	1995	57	49	86.0	24	151	88	No	Yes
Mulcahy [43]	1996	15	15	100	24			Yes	Yes
Ure [12]	1998	20	20	100	42	205.2	118.8	Yes	Yes
Callaghan [11]	1999	12	10	83.3	120			Yes	Yes
Oussedik [44]	2010	11	11	100	66	105.7	81.6	Yes	Yes
Klouche [45]	2012	38	38	100	24	61	35	No	No
Zeller [10]	2014	157	149	94.9	28.1	66.9	41.6	Yes	No
Jenny [46]	2014	65	55	84.6	36	72		Yes	Yes
Total		1208	1005		-	-	-		
Minimum		11	-	75	24.0	61.0	35.0		
Maximum		583	-	100.0	120.0	205.2	118.8		
Mean		39.5	-	83.2	40.2	109.5	66.6		
SD *		38.8	-	-	27.9	46.9	30.4		

* SD: Standard deviation.

**Table 2:** Data from studies reporting cementless one-stage hip revision surgery for delayed (>6 weeks after surgery) peri-prosthetic hip infection at a minimum follow-up of 24 months.

Author	Year	Number of Patients	Number of Patients Free from Infection at Follow-Up	Percent of Patients Free from Infection at Follow-Up	Follow-Up (Months)			Selection Bias	Local Antimicrobial Protection
					Min	Max	Mean		
Garcia [47]	2004	7	7	100	24			No	No
Rudelli [48]	2008	32	30	93.8	63	183	103	Not reported	No
Winkler [49]	2008	37	34	91.9	24	96	52.8	Yes	Antibiotic-loaded allografts
Yoo [50]	2009	12	10	83.3	39.6	135.6	86.4	Yes	No
Wolf [51]	2014	37	21	56.8	24			No	No
Bori [52]	2014	24	23	95.8	25	94	45	Yes	No
Li [53]	2015	6	6	100.0	78	187.2	103.2	Yes	No
Born [54]	2016	28	28	100.0	24	180	84	Yes	No
Ebied [55]	2016	33	32	97.0	48	96	60	Yes	Antibiotic-loaded allografts
Whiteside [56]	2017	21	20	95.2	25	157	63	No	Intra-articular antibiotic infusion
Lange [57]	2018	56	51	91.1	24		48	No	Gentamicin collagen fleece
Capuano [58]	2018	5	5	100.0	24	36	29.3	No	Antibiotic-loaded hydrogel coating
Ji [59]	2019	111	99	89.2	24	107	58	No	Vancomicin or Imipenem powder and intra-articular antibiotic infusion
Pellegrini [60]	2021	10	10	100.0	24	60	37.2	Yes	Antibiotic-loaded hydrogel coating
Ji [61]	2022	29	26	89.7	24	133	85	No	Intra-articular antibiotic infusion
Dersch [62]	2022	38	35	92.1	24	187.2	67.2	Yes	Antibiotic-loaded allografts
Mangin [63]	2023	35	32	91.4	24	132	60	Yes	No
Total		521	469		-	-	-		
Minimum		5	-	56.8	24.0	36.0	29.3		
Maximum		111	-	100.0	78	187.2	103.2		
Mean		30.6	-	90.0	31.9	127.4	65.5		
SD *		25.0	-	-	16.2	48.4	22.5		

* SD: Standard deviation.

**Table 3:** Local antibacterial protection and infection recurrence for one-stage cementless hip revision: pooled results.

Local Antibacterial Protection	Number of Patients	Number of Patients Free from Infection at Follow-Up	Percent of Patients Free from Infection at Follow-Up (Mean, Min, Max)	Follow-Up (Months) (Mean and SD *)
None [47,48,50,51,52,53,54,63]	181	157	86.7 (56.8–100)	80.3 ± 23.4
Intra-articular antibiotic infusion or local antibiotic vancomicin powder [56,59,61]	161	145	90.1 (89.2–95.2)	68.7 ± 14.4
Gentamicin-loaded collagen fleece [57]	56	51	91.1	48
Antibiotic-loaded allografts [49,55,62]	108	101	93.5 (91.9–92.1)	60.0 ± 7.2
Antibiotic-loaded hydrogel coating [58,60]	15	15	100.0	30.1 ± 6.8

* SD: Standard deviation.

## Data Availability

Not applicable.
